# Supplementary figures and images for: Inhibition of vaccinia virus L1 N-myristoylation by the host N-myristoyltransferase inhibitor IMP-1088 generates non-infectious virions defective in cell entry
Source: PLoS Pathog. 2022 Oct 10;18(10):e1010662. doi: 10.1371/journal.ppat.1010662 (PMC9584500; doi:10.1371/journal.ppat.1010662)

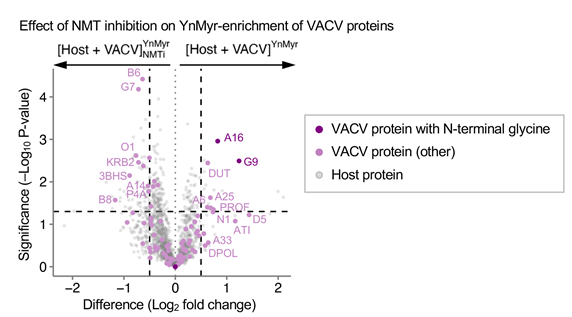

Supplement: S1 Fig — Target engagement of NMT inhibitor (NMTi = IMP-1088) on VACV proteins in infected host cells. The horizontal and vertical axes show the difference in quantified protein levels and the significance of the quantified difference, respectively. The labels above the right and left arrow indicate the two conditions being compared within the plot. The dashed vertical lines depict -0.5 Log2 and +0.5 fold change; horizontal dashed line depicts significance cut-off (p = 0.05).Left vertical line depicts -0.5 Log2 fold change, right of +0.5; horizontal line depicts significance cut-off (P = 0.05); 3 N-myristoylated VACV proteins depicted in purple, 115 VACV proteins in pink, 2738 human proteins in gray. (TIF) [file ppat.1010662.s002.tif]

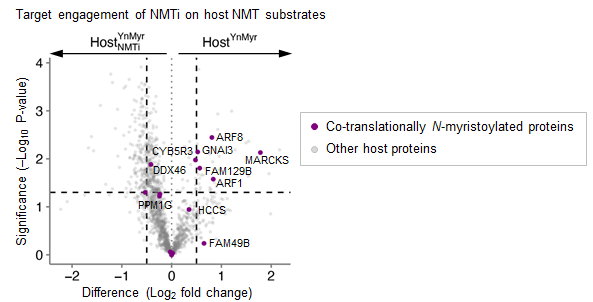

Supplement: S2 Fig — Target engagement of NMT inhibitor (NMTi = IMP-1088) in host cells, as visualized by the effect on 32 known co-translationally N-myristoylated proteins of the host (purple). Other proteins in gray. The horizontal and vertical axes show the difference in quantified protein levels and the significance of the quantified difference, respectively. The labels above the right and left arrow indicate the two conditions being compared within the plot. The dashed vertical lines depict -0.5 Log2 and +0.5 fold change; horizontal dashed line depicts significance cut-off (p = 0.05). Left vertical line depicts -0.5 Log2 fold change, right of +0.5; horizontal line depicts significance cut-off (P = 0.05). Other proteins in gray. (TIF) [file ppat.1010662.s003.tif]

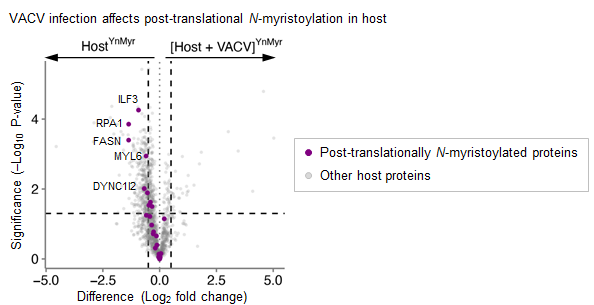

Supplement: S3 Fig — Effect of VACV infection on 27 known post-translationally N-myristoylated protein levels of the host (purple). Other proteins in gray. The horizontal and vertical axes show the difference in quantified protein levels and the significance of the quantified difference, respectively. The labels above the right and left arrow indicate the two conditions being compared within the plot. The dashed vertical lines depict -0.5 Log2 and +0.5 fold change; horizontal dashed line depicts significance cut-off (p = 0.05). Left vertical line depicts -0.5 Log2 fold change, right of +0.5; horizontal line depicts significance cut-off (P = 0.05). Other proteins in gray. (TIF) [file ppat.1010662.s004.tif]
